# Psychometric Properties of the Problem Areas in Diabetes (PAID) Instrument in Singapore

**DOI:** 10.1371/journal.pone.0136759

**Published:** 2015-09-03

**Authors:** Kavita Venkataraman, Luor Shyuan Maudrene Tan, Dianne Carrol Tan Bautista, Konstadina Griva, Yasmin Laura Marie Zuniga, Mohamed Amir, Yung Seng Lee, Jeannette Lee, E. Shyong Tai, Eric Yin Hao Khoo, Hwee Lin Wee

**Affiliations:** 1 Saw Swee Hock School of Public Health, National University of Singapore and National University Health System, Singapore, Singapore; 2 Center for Quantitative Medicine, Duke NUS Graduate Medical School, Singapore, Singapore; 3 Singapore Clinical Research Institute, Singapore, Singapore; 4 Department of Psychology, National University of Singapore, Singapore, Singapore; 5 Department of Medicine, Yong Loo Lin School of Medicine, National University of Singapore, Singapore; 6 Department of Paediatrics, Yong Loo Lin School of Medicine, National University of Singapore, Singapore; 7 Khoo Teck Puat-National University Children's Medical Institute, National University Hospital, Singapore, Singapore; 8 Singapore Institute for Clinical Sciences, A*STAR, Singapore, Singapore; 9 Department of Pharmacy, National University of Singapore, Singapore, Singapore; Swinburne University of Technology, AUSTRALIA

## Abstract

**Background:**

Emotional distress is an important dimension in diabetes, and several instruments have been developed to measure this aspect. The Problem Areas in Diabetes (PAID) scale is one such instrument which has demonstrated validity and reliability in Western populations, but its psychometric properties in Asian populations have not been examined.

**Methods:**

This was a secondary analysis of data from patients with Type 2 diabetes mellitus recruited through convenience sampling from a diabetes specialist outpatient clinic in Singapore. The following psychometric properties were assessed: Construct validity through confirmatory factor analysis (CFA) and Rasch analysis, concurrent validity through correlation with related scales (Kessler Psychological Distress Scale, Diabetes Health Profile—psychological distress, Audit of Diabetes Dependent Quality of Life), reliability through assessment of internal consistency and floor and ceiling effects, and sensitivity by estimating effect sizes for known clinical and social functioning groups.

**Results:**

203 patients with mean age of 45±12 years were analysed. None of the previously published model structures achieved a good fit on CFA. On Rasch analysis, four items showed poor fit and were removed. The abridged 16-item PAID mapped to a single latent trait, with a high degree of internal consistency (Cronbach ɑ 0.95), but significant floor effect (24.6% scoring at floor). Both 20-item and 16-item PAID scores were moderately correlated with scores of related scales, and sensitive to differences in clinical and social functioning groups, with large effect sizes for glycemic control and diabetes related complications, nephropathy and neuropathy.

**Conclusion:**

The abridged 16-item PAID measures a single latent trait of emotional distress due to diabetes whereas the 20-item PAID appears to measures more than one latent trait. However, both the 16-item and 20-item PAID versions are valid, reliable and sensitive for use among Singaporean patients with diabetes.

## Introduction

The Problem Areas in Diabetes (PAID) instrument was developed to measure emotional distress in people with diabetes. It is a 20-item scale consisting of emotional problems commonly reported in type 1 and type 2 diabetes mellitus, and has been found to be a valid and reliable scale in Western populations[1–3]. It has also been found to be responsive, that is, able to detect change when used in intervention studies[4]. Several language versions of the scale have also been tested and found to be valid and reliable for use in the specific populations[3, 5–8]. The original scale was constructed as a single domain structure, with an underlying emotional distress factor being related to all the items[1]. Subsequently, other researchers have found conflicting results, with some confirming the original single-factor structure[2, 8], and others identifying multiple sub-dimensions[3, 6, 7].

Type 2 diabetes mellitus is becoming an important public health problem in Singapore, with a prevalence of 11.3% in 2010[9], and a projected prevalence of 15% by 2050[10]. Several patient reported outcome measures have been examined for their validity in this patient population. As emotional distress is an important dimension in diabetes and affects not only the patient’s experience of disease and care, but also their compliance with treatment and lifestyle regimens[11, 12], it will be useful to examine the psychometric properties of the PAID scale in this context. While a Chinese translation of the PAID instrument has been validated in Taiwan and found to have a single-factor structure[8], the original English version has not been similarly examined in any Asian context. In this study, we attempt to evaluate the validity, both construct and concurrent, reliability and sensitivity of PAID in a group of Singaporean patients with Type 2 diabetes mellitus.

## Methods

This is a secondary analysis of the baseline data of a prospective longitudinal study on outcomes in patients with diabetes mellitus (PEAQ DM). The study recruited patients aged between 21 and 65 years old, who were diagnosed with diabetes (both Type 1 and Type 2) for at least one year. The upper age limit was set at 65 years as older people are more likely to have other associated comorbid conditions that could affect the level of emotional distress. Patients were recruited at least one year post-diagnosis to avoid confounding of findings by any short-term increase in anxiety and stress due to diabetes diagnosis, as suggested by previous research[13, 14]. Patients undergoing routine clinic visits at the specialist Endocrinology outpatient clinic of the National University Hospital were selected by convenience sampling at the clinic waiting area from 2011 to 2012. Only English literate patients were included in the study. Patients were excluded if there was self-reported or documented unstable and ongoing treatment of heart, kidney, liver and psychiatric conditions. This study was approved by the National Healthcare Group Domain Specific Review Board (Protocol No.: 2011/02018), and written informed consent obtained from all patients prior to participation. Only patients with type 2 diabetes were included in this analysis.

### Demographics

Demographic details such as age, gender, ethnicity, educational status, marital status, type of housing and household income were collected using self-administered questionnaires. Ethnicity was classified as Chinese, Malay, Asian Indian or Others. Marital status was classified as “never married”, “currently married” or “separated/divorced/widowed”. Education level was categorized into <7, 7–10 and >10 years of schooling. Housing was categorized into Housing Development Board flat (HDB) of 4 rooms or smaller, 5-room HDB/ Executive flat and private housing, representing increasing socioeconomic status. Monthly household income was categorised as less SGD 4000, SGD 4000–7999, and SGD 8000 and above, where SGD 7999 corresponds to the median household income[15].

### PAID

PAID is a self-administered 20-item scale. Each item is scored from 0 (not a problem) to 4 (serious problem). The sum of all item scores multiplied by 1.25 gives the total PAID score, which ranges from 0 to 100, higher scores reflecting greater emotional distress. A score of 40 or above is indicative of severe emotional distress [16].

### Other psychological scales

The Kessler Psychological Distress Scale (K10) is a 10-item global measure of distress, with questions on anxiety and depressive symptoms in the past four weeks. All items are scored on a scale of 1 (none of the time) to 5 (all of the time). The sum of all item scores yields the total score, which has a range of 10 to 50[17].

The Diabetes Health Profile consists of 18 items and three sub-scales: psychological distress (DHP-PD), barriers to activity and disinhibited eating. The 6-item DHP-PD sub-scale was used to correlate PAID scores in this analysis. Each item was scored on a scale of 0 to 3. The sum of the 6 items divided by 18 and then multiplied by 100, gave the DHP-PD sub-scale score[18].

The Audit of Diabetes Dependent Quality of Life (ADDQoL) is a 19-item scale measuring diabetes-specific quality of life. Respondents rate the impact of diabetes on a domain from -3 (maximum negative impact) to +3 (maximum positive impact), and the importance of that domain on a scale of 0 (not important) to 3 (very important). The impact and importance ratings are multiplied to give the score for that domain. These scores are averaged across applicable domains to derive the overall score[19]. Scores range from -9 to +3, with lower scores reflecting poorer QoL.

All scales were scored according to their respective manuals.

### Known groups

#### Clinical

Glycemic control was determined by glycated haemoglobin (HbA1c), and classified as good control (HbA1c ≤ 7.0%) and poor control (HbA1c > 7.0%). HbA1c was retrieved from the electronic medical record and is routinely measured at the National University Hospital Referral Laboratory, which is accredited by the College of American Pathologists using an assay accredited by the National Glycoprotein Standardization Program with controls traceable to the Diabetes Control and Complications Trial (DCCT). Medical history of co-morbidities and complications (cardiovascular disease, retinopathy, nephropathy, peripheral vascular disease, cerebrovascular disease and anaemia) were captured through a combination of self-report and electronic medical record search.

#### Social functioning

Patients were asked to rate their effectiveness at work and outside of work on a scale of 0–10. This was dichotomised into effective (6–10) and not effective (0–5) for both at work and outside. Satisfaction with family life was recorded on a single-item with Likert scale ranging from poor to excellent. The scores were subsequently collapsed into not satisfied (poor and fair) and satisfied (good, very good, excellent) with family life.

### Statistical analysis

Means and SDs were used to describe continuous variables, while counts and proportions were used for categorical variables. For purposes of analysis, age was stratified into those below 45 years of age and those 45 years and above, 45 being the mean age of the group. All analyses were performed using STATA version 11 (StataCorp LP), except calculation of effect sizes for sensitivity analysis, which were computed in MS Excel.

#### Construct validity

Construct validity is the degree to which a test measures what it is designed to measure. Factor analysis is an accepted method to assess construct validity[20]. We used confirmatory factor analyses (CFA) based on all published factor structures[3, 5–7, 21–23]. A comparative fit index (CFI) and Tucker Lewis index (TFI) of more than 0.9 were taken as indicative of a good fit. Interestingly, none of the models based on previously published factors achieved a good model fit. Only the single factor structure and Miller et al’s[22] two-factor structure achieved model convergence, with CFI and TFI of 0.76 and 0.74, and 0.79 and 0.76, respectively. As such, we used Rasch analysis [24] to evaluate if the 20 PAID items measure a single latent variable (diabetes-related emotional distress). The items were recoded into dichotomous variables with response levels 0 and 1 combined as “No or mild problem” and response levels 2 to 4 combined as “Moderate to severe problem” to facilitate Rasch analysis. As the latent trait does not follow a normal distribution, conditional maximum likelihood was used to estimate the difficulty parameter. Items with Infit or Outfit values exceeding +/-2 were regarded to have poor model fit and were excluded from the model. Infit relates to unexpected behaviour affecting responses to items that are near the person ability level whereas outfit relates to unexpected behaviour affecting responses to items that are further away from the person ability level. The analysis was repeated with the misfitting items removed until there was no more item with infit or outfit value exceeding +/-2. The item-person map was generated to evaluate if the difficulty range of the items adequately covered the ability range of the persons. In this case, item difficulty refers to the level of diabetes-related emotional distress needed for an individual to endorse a particular item while person ability refers to the level of diabetes-related emotional distress experienced by an individual.

#### Concurrent validity

16-item PAID scores were compared with scores of other scales measuring similar or related constructs: K10, DHP-PD and ADDQOL using Spearman correlations.

#### Reliability

Internal consistency of the scale was assessed by Cronbach’s alpha coefficient[25]. The percentage of respondents scoring at the floor (total score = 0) and ceiling (total score = 100) was also determined. A floor (ceiling) effect was defined as being present if >15% of the subjects scored at the minimum (maximum) level respectively[26].

#### Sensitivity

Sensitivity is the ability of an instrument to detect a difference between patient sub-groups that is both clinically relevant and statistically significant[27–29]. Sensitivity was determined by computing the effect size (difference in mean scores/ pooled standard deviation) for the known demographic, clinical and social functioning groups as described above. Effect sizes of 0.2, 0.5 and 0.8 were considered small, moderate and large, respectively[30]. One-way ANOVA was used to test for significant differences in PAID scores between these sub-groups.

## Results

**Fig 1:**
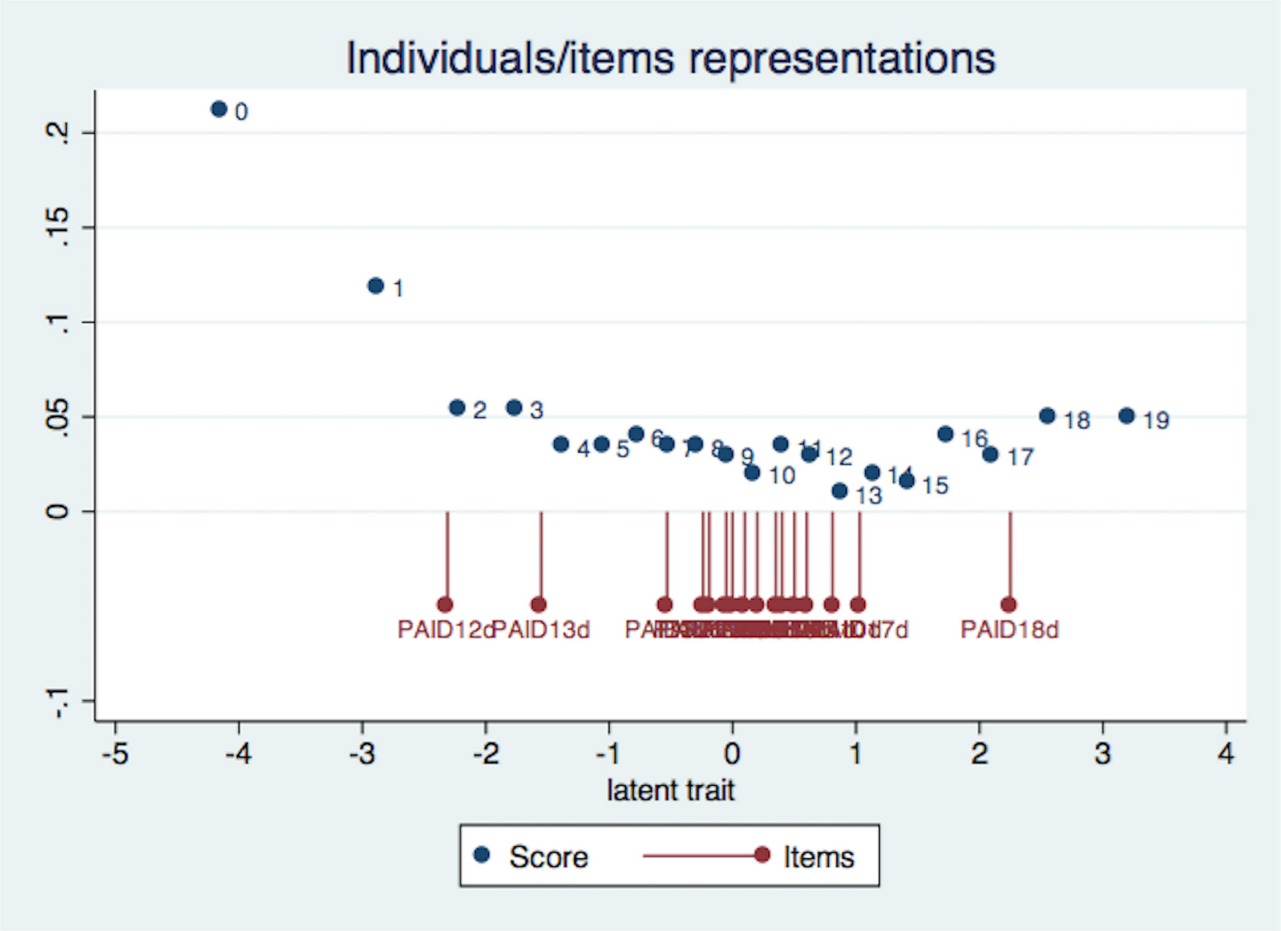
Item-person map illustrating the distribution of item difficulty along the y-axis and person ability along the x-axis.

Of the 578 patients approached for participation, 185 declined, while another 89 did not meet the inclusion criteria. Of the 304 patients recruited, 82 had type 1 diabetes. Five of the 222 patients with type 2 diabetes did not complete study procedures, and therefore data was available for only 217 patients. Of these, 10 had PAID items missing while 4 had DHP-PD items missing, and were excluded. The remaining 203 patients were included in the analysis. The mean age of the patients was 45 years, with 64% (130) men (Table 1). The majority were Chinese, with over seven years of education, and married. Mean PAID score was 28.8(±21.9), with 65 (32%) reporting a PAID score of 40 or above, denoting severe emotional distress. The difficulty range of the items provided adequate coverage of the ability range of the persons (Fig 1).

**Table 1:** Characteristics of participants in the study (N = 203).

Characteristic	N	%
Gender		
Male	130	64.0
Female	73	36.0
Ethnicity		
Chinese	103	50.7
Malay	23	11.3
Indian	56	27.6
Others	21	10.3
Education		
< 7 yrs	15	8.0
7–10 yrs	65	34.6
> 10 yrs	108	57.5
Marital status		
Single	41	21.9
Married	126	67.4
Divorced/Widowed	20	10.7
Housing type		
1–4 room HDB	88	47.8
5 room HDB/ exec	61	33.2
private housing	35	19.0
Co-morbidities (yes)		
Retinopathy	25	15.6
Cardiovascular Disease	25	12.3
Nephropathy	16	9.4
Neuropathy	14	8.3
Cerebrovascular Disease	12	5.9
Anemia	12	7.1
PVD	6	3.4
	**Mean(SD)**	**95% CI**
Age	45 (11.9)	43.7–47
Mean HbA1c	8.3 (1.9)	8.0–8.5
Pyschological Distress scales		
PAID	28.8 (21.9)	25.8–31.8
K10	19.4 (6.9)	18.4–20.3
DHP-PD	21.3 (23.2)	18.2–24.4
ADDQoL	-2.9 (2.2)	-3.2–-2.6

### Construct validity

Four items (*Not having clear and concrete goals for diabetes care*, *Feeling depressed when thinking about living with diabetes*, *Feeling that diabetes is taking up too much of mental and physical energy every day and Feeling that friends and family are not supportive of diabetes management efforts*) were removed because of misfit (Table 2). The remaining 16 items, henceforth referred to as 16-item PAID, provided good coverage of item difficulty. 69 out of 203 subjects (34%) achieved full scores on the 16-item PAID.

**Table 2:** Item difficulty and fit statistics from Rasch analysis.

Items	Difficulty Parameters	Std. Err.	R1c	df	p-value	Standardized Outfit	Standardized Infit	U
**PAID1. no clear goals for care**	**-0.525**	**0.310**	**22.447**	**2**	**0**	**3.846**	**4.720**	**4.110**
PAID2. discouraged with treatment plan	0.397	0.316	2.033	2	0.3618	-1.194	-0.600	-1.185
PAID3. feel scared about living with diabetes	-0.525	0.310	1.984	2	0.3709	-1.830	-1.663	-1.564
PAID4. uncomfortable social situations relating to diabetes	0.500	0.317	1.000	2	0.6066	-0.882	-0.750	-0.481
PAID5. feel deprived about food	0.098	0.313	0.510	2	0.7748	0.282	0.437	0.472
**PAID6. feel depressed about living with diabetes**	**0.197**	**0.314**	**5.139**	**2**	**0.0766**	**-2.036**	**-2.630**	**-1.317**
PAID7. not knowing if moods related to diabetes	-0.049	0.312	0.510	2	0.775	0.947	0.596	1.414
PAID8. feel overwhelming by diabetes	0.347	0.315	5.714	2	0.0575	-1.865	-1.476	-2.199
PAID9. worry about low blood sugar reactions	0.098	0.313	2.348	2	0.3091	0.988	1.779	0.949
PAID10. feel angry about living with diabetes	0.815	0.321	2.165	2	0.3387	-1.677	-1.518	-1.019
PAID11. feel constantly concerned about eating	-0.241	0.311	1.414	2	0.4932	0.192	1.203	0.346
PAID12. worrying about the future	-2.307	0.322	1.388	2	0.4996	0.227	0.413	1.427
PAID13. feeling guilty when off track with diabetes management	-1.551	0.313	0.643	2	0.7252	0.137	1.055	1.714
PAID14. not accepting diabetes	0.603	0.318	2.144	2	0.3423	-1.732	-1.741	-0.992
PAID15. feel unsatisfied with physician	2.247	0.349	1.295	2	0.5234	0.179	-0.822	0.294
**PAID16. feel that diabetes takes up too much energy**	**0.397**	**0.316**	**5.533**	**2**	**0.0629**	**-2.382**	**-2.244**	**-1.742**
PAID17. feel alone with diabetes	1.033	0.324	2.247	2	0.3251	-0.754	-1.828	-1.006
**PAID18. feel family not supportive of diabetes management efforts**	**2.247**	**0.349**	**5.564**	**2**	**0.0619**	**0.244**	**2.226**	**1.558**
PAID19. coping with diabetes complications	-0.193	0.311	3.586	2	0.1665	0.536	1.393	1.232
PAID20. feel burned out by effort needed to manage diabetes	0.000	.	7.057	2	0.0294	-1.490	-1.683	-0.636

R1c test R1c = 73.481 38 0.0005.

Andersen LR test Z = 77.248 38 0.0002.

Items with infit or outfit statistics exceeding +/-2 are in bold.

### Concurrent validity

16-item PAID scores were moderately correlated with K10 (rho 0.53, *p* <0.001), DHP-PD (rho 0.56, *p* <0.001) and ADDQoL (rho -0.54, *p* <0.001).

### Reliability

Cronbach’s alpha was 0.95 for the 16-item PAID, indicating a high degree of internal consistency. There was a significant floor effect using the revised scale, with 24.6% of the respondents scoring at the floor. 9.4% scored at the ceiling. This was in contrast to the original scale where there were no significant floor or ceiling effects, with 5.4% of respondents scoring at the floor and none at the ceiling.

### Sensitivity

The PAID scale did not distinguish between patients from different socio-demographic groups, except for education, household income and housing type, which had small to moderate effect sizes (Table 3). On the other hand, the scale was able to discriminate well between clinical groups, with moderate to large effect sizes for glycemic control and complication groups. The largest effect sizes were for glycemic control (1.07 for HbA1c > 8% with HbA1c <7% as reference), and nephropathy (1.02) and neuropathy (0.8) compared to those with no complications. PAID was also able to distinguish between those with greater effectiveness at work and outside, but the effect sizes were small to moderate.

**Table 3:** Comparison of 16-item PAID scores across known demographic, clinical and social functioning groups.

Variable^1^	N	Mean	Std. Dev.	Effect size	*P* ^2^
Clinical groups					
Glycemic control					
Hba1c = <7.0	50	3.1	4.3		
Hba1c 7–8	63	4.8	5.4	0.35	0.257
Hba1c>8.0	90	8.7	5.7	**1.07**	**<0.001**
Complications					
No complications	30	4.8	4.9		
Retinopathy	25	7.3	5.1	**0.49**	**0.043**
Cardiopathy	25	7.9	6.2	**0.56**	**0.052**
Nephropathy	16	10.0	5.5	**1.02**	**0.002**
Neuropathy	14	8.9	5.7	**0.80**	**0.013**
Cerebro-vascular problems	12	7.8	6.3	0.57	0.146
Anaemia	12	5.3	4.8	0.10	0.701
Social functioning groups					
Effectiveness at work					
No	24	8.3	6.3		
Yes	167	5.6	5.6	**-0.47**	**0.032**

^1^ –Additional variables tested with non-significant differences in PAID scores–Socio-demographic groups (age, gender. Ethnicity, marital status, education, housing type, income); Clinical groups (diabetes duration); Social functioning groups (effectiveness outside work, family life satisfaction).

^2 –^oneway ANOVA with Bonferroni corrections when multiple comparisons were made.

## Discussion

Consistent with the CFA, Rasch analysis revealed that PAID does not measure a single factor among Singaporeans with Type 2 diabetes. This was also reported in other studies. A four-factor solution fit the PAID scale best in a population of US and Dutch patients with diabetes[3], and the Norwegian version[5], while three- and two-factor solutions best fit the Swedish[6] and Icelandic[7] versions. However, if the misfitting items were removed, the 16-item PAID will measure a single latent construct of diabetes-related emotional distress with a minimum score of 0 and maximum score of 16. The advantage of using the Rasch model is that it is an interval scale. Similar to other researchers, we found a high degree of internal consistency [2, 3, 5–7] with the original PAID as well as the 16-item PAID, though there appeared to be a significant floor effect with the 16-item PAID.

The scale also showed moderate correlations with other measures having related constructs, indicating reasonable concurrent validity, and were similar to what has been previously reported[3, 5, 21].

The 16-item PAID appeared to be a sensitive instrument, able to distinguish between clinically important groups. We were able to demonstrate a large effect size between those having good versus poor glycemic control. This is in line with existing literature, with weak to moderate correlations reported with HbA1c in cross-sectional studies[2, 5, 7], and small to moderate effect sizes in intervention studies[4]. There is little literature on the sensitivity of PAID with respect to diabetes-related complications, but we have demonstrated that the scale is sensitive to complication status as well. PAID scores did not vary with socio-demographic characteristics except income and housing type, which showed statistically non-significant differences. This demonstrates the instrument’s consistency across various age, ethnic, gender and social groups.

As it was not our intention at the outset to reduce the number of items, we conducted further analyses using the original 20-item PAID. The results of the comparison are given in the Appendix (S1 Appendix). The findings were very similar except that there is no floor effect with the original scale and the original scale was able to discriminate between patients with poor and good family life satisfaction.

We believe that we have added new information to the literature as this is the first study to apply Rasch analysis to PAID, and this has not been reported previously to our knowledge (based on PubMed search using keywords “PAID” [ti/abs] AND diabetes [ti/abs] AND rasch). Other strengths of our study are a multi-ethnic population of patients and a socio-cultural context where the psychometric properties of PAID have not been previously assessed. We have followed this up with an evaluation of the sensitivity of the instrument to relevant clinical groups, and specifically complications, which have not been reported before. There are also certain limitations, chiefly the cross-sectional nature of the study, which precluded assessment of the test-retest reliability and responsiveness of the instrument, and the inclusion of only English-speaking patients in the study. While this may limit generalizability of our findings, almost 80% of the population is English-literate[31] so the findings will be applicable to the majority. Other factors that may limit study generalizability are the exclusion of older people with diabetes, and disproportionate representation of males (two-thirds) in our sample.

## Conclusion

The abridged 16-item PAID measures a single latent trait of emotional distress due to diabetes whereas the 20-item PAID appears to measure more than one latent trait. However, both versions are valid, reliable and sensitive for use among Singaporean patients with diabetes. In fact, the 20-item PAID has slightly better psychometric properties in that it does not exhibit floor effects and can discriminate better. We would recommend keeping to the original 20-item PAID as this would allow for the scores to be compared with other international studies. Clinicians, case managers as well as researchers interested in assessing diabetes-related distress would now have a valid and reliable instrument to use as an outcome measures for interventions in clinical care and research.

## Supporting Information

S1 Appendix Concurrent validity and sensitivity of 20-item PAID. (DOCX)
