# Supplementary material for: Psychometric Properties of the Problem Areas in Diabetes (PAID) Instrument in Singapore
Source: PLoS One. 2015 Sep 3;10(9):e0136759. doi: 10.1371/journal.pone.0136759 (PMC4559380; doi:10.1371/journal.pone.0136759)
Supplement: S1 Appendix — (DOCX) [file pone.0136759.s001.docx]

**S1 Appendix. Concurrent validity and sensitivity of 20-item PAID.**

**Table S.1 Concurrent validity of 20-item PAID scores by comparison with scores from related scales**

| Scale | Spearman’s Rho | *P* |
| --- | --- | --- |
| Kessler Psychological Distress Scale | 0.56 | <0.001 |
| Diabetes Health Profile- psychological distress | 0.6 | <0.001 |
| Audit of Diabetes Dependent Quality of Life | -0.62 | <0.001 |

**Table S.2.** **Comparison of 20-item PAID scores across known demographic, clinical and social functioning groups.**

| Variable | N | | Mean | S.D. | Effect size | *P* |
| --- | --- | --- | --- | --- | --- | --- |
| **Socio-demographic groups** | | | |  |  |  |
| ***Age*** |  | |  |  |  |  |
| **Age<45** | 87 | | 31.15 | 21.22 |  |  |
| **Age>=45** | 115 | | 26.55 | 21.75 | -0.21 | 0.136 |
| ***Gender*** |  | |  |  |  |  |
| **Male** | 130 | | 28.01 | 21.18 |  |  |
| **Female** | 73 | | 30.22 | 23.16 | 0.10 | 0.491 |
| ***Ethnicity*** |  | |  |  |  |  |
| **Chinese** | 103 | | 28.79 | 20.44 |  |  |
| **Malay** | 23 | | 32.77 | 26.35 | 0.18 | 1.000 |
| **Indian** | 56 | | 26.70 | 22.01 | -0.10 | 1.000 |
| **Others** | 21 | | 30.18 | 24.01 | 0.07 | 1.000 |
| ***Marital status*** | | |  |  |  |  |
| **Single** | 41 | | 29.36 | 17.62 | 0.02 |  |
| **Married** | 126 | | 28.84 | 23.16 |  | 1.000 |
| **Widow/separated** | 20 | | 29.06 | 22.47 | 0.01 | 1.000 |
| ***Education*** | | |  |  |  |  |
| **<7 yrs** | 15 | | 27.00 | 23.73 |  |  |
| **7-10 yrs** | 65 | | 33.75 | 23.29 | 0.29 | 0.859 |
| **>10 yrs** | 108 | | 26.56 | 21.02 | -0.02 | 1.000 |
| ***Housing type*** | | |  |  |  |  |
| **1-4 room** | 88 | | 31.12 | 22.22 |  |  |
| **5room/ exec** | 61 | | 31.91 | 23.27 | 0.04 | 1.000 |
| **Private** | 35 | | 19.39 | 18.73 | -0.59 | 0.025 |
| ***Income (SGD/month)*** |  | |  |  |  |  |
| **<4000** | 74 | | 33.40 | 22.36 |  |  |
| **4000-7999** | 50 | | 25.35 | 19.33 | -0.41 | 0.121 |
| **8000 and above** | 38 | | 25.99 | 21.40 | -0.34 | 0.247 |
| **Clinical groups** | | |  |  |  |  |
| ***Diabetes duration*** | |  |  |  |  |  |
| ≤ 5 years | | 80 | 27.25 | 21.52 |  |  |
| 6 -10 years | | 44 | 31.14 | 23.64 | 0.17 | 1.000 |
| > 10 years | | 57 | 28.82 | 21.31 | 0.07 | 1.000 |
| ***Glycemic control*** | | |  |  |  |  |
| **Hba1c <7.0** | 50 | | 18.53 | 17.90 |  |  |
| **Hba1c 7.0-8.0** | 63 | | 22.46 | 18.78 | 0.22 | 0.897 |
| **Hba1c>8.0** | 90 | | 38.96 | 21.74 | 1.00 | <0.001 |
| ***Complications*** | | |  |  |  |  |
| **No complications** | 30 | | 24.00 | 19.27 |  |  |
| **Retinopathy** | 25 | | 33.70 | 20.31 | 0.49 | 0.044 |
| **Cardiopathy** | 25 | | 38.15 | 25.94 | 0.63 | 0.021 |
| **Nephropathy** | 16 | | 44.14 | 21.40 | 1.01 | 0.002 |
| **Neuropathy** | 14 | | 39.91 | 21.36 | 0.80 | 0.012 |
| **Cerebro** | 12 | | 36.88 | 21.78 | 0.64 | 0.094 |
| **Anaemia** | 12 | | 25.94 | 16.04 | 0.11 | 0.671 |
| **Social functioning groups** | | | |  |  |  |
| ***Effectiveness at work*** | | | |  |  |  |
| **No** | 24 | | 35.47 | 24.09 | 0.33 | 0.075 |
| **Yes** | 167 | | 27.24 | 20.63 |  |  |
| ***Effectiveness outside work*** | | | |  |  |  |
| **No** | 50 | | 32.58 | 24.75 | 0.21 | 0.136 |
| **Yes** | 144 | | 27.31 | 20.19 |  |  |
| ***Family life satisfaction*** | | | |  |  |  |
| **Poor** | 21 | | 33.69 | 21.34 | 0.24 | 0.297 |
| **Good** | 179 | | 28.40 | 21.99 |  |  |
